# Identification of immune characteristic biomarkers and therapeutic targets in cuproptosis for rheumatoid arthritis by integrated bioinformatics analysis and single-cell RNA sequencing analysis

**DOI:** 10.3389/fmed.2025.1520400

**Published:** 2025-03-17

**Authors:** Xianbin Li, Xueli Zhang, Tao Liu, Guodao Zhang, Dan Chen, Suxian Lin

**Affiliations:** ^1^School of Computer and Big Data Science, Jiujiang University, Jiujiang, China; ^2^Department of Digital Media Technology, Hangzhou Dianzi University, Hangzhou, China; ^3^Jiujiang Key Laboratory of Digital Technology, Jiujiang, China; ^4^Department of Medical Technology, Zhengzhou Railway Vocational and Technical College, Zhengzhou, China; ^5^Department of Rheumatology, The First Affiliated Hospital of Wenzhou Medical University, Zhejiang, China; ^6^Department of Rheumatology, Wenzhou People’s Hospital, Wenzhou, China

**Keywords:** rheumatoid arthritis, cuproptosis, immune infiltration, therapeutic drugs, single-cell RNA

## Abstract

**Introduction:**

Rheumatoid arthritis (RA) is a chronic autoimmune disorder intricately liked with inflammation. Cuproptosis, an emerging type of cell death, has been implicated in the initiation and development of RA. However, the exact alterations in the expression and biological function of cuproptosis-related genes (CRGs) in RA remain poorly understood. Therefore, our study aims to elucidate the potential association between CRGs and RA, with the goal of identifying novel biomarkers for the treatment and prognosis of RA.

**Methods:**

In this study, we identified ten differentially expressed cuproptosis-related genes (DE-CRGs) between patients with RA and controls. Through comprehensive functional enrichment and protein-protein interaction (PPI) network analysis, we explored the functional roles of the DE-CRGs. Additionally, we investigated the correlation between DE-CRGs and immune infiltration, immune factors, diagnostic efficacy, and potential therapeutic drugs.

**Results:**

Leveraging single-cell RNA sequencing data, we conducted a detailed analysis to elucidate alterations in various cell clusters associated with RA. Our study unveiled a significant association between DE-CRGs and diverse biological functions, as well as potential drug candidates.

**Discussion:**

These findings provide crucial insights into the involvement of DE-CRGs in the pathogenesis of RA and shed light on potential therapeutic strategies.

## Introduction

Rheumatoid arthritis (RA) is a chronic autoimmune disease intricately linked to inflammation. Recent statistics reveal a global prevalence of RA as high as 0.24%, with an annual incidence ranging from 20 to 45 per 100,000 individuals (1). In existing guidelines, the diagnosis of RA is contingent upon clinical manifestations, physical examination findings, as well as serological and imaging results (2). Regrettably, while most patients with RA exhibit positive laboratory test results (rheumatoid serum factor and ACPA), approximately one-third of RA cases yield negative findings (3). Furthermore, no single pathological laboratory discovery or imaging method has been demonstrated to definitively diagnose RA, posing significant challenges to its diagnosis and subsequently influencing treatment and prognosis. Moreover, numerous studies emphasize the importance of early diagnosis, prompt intervention, timely treatment initiation, and, if necessary, immediate referrals for RA patients (4–6). Despite advancements, RA remains incurable, and disease-modifying anti-rheumatic drugs (DMARDs) represent the primary treatment option (7). Nevertheless, prolonged DMARD use is often associated with adverse effects, including gastric ulcers, vomiting, heartburn, or gastrointestinal bleeding (8, 9). Hence, the imperative for dependable biomarkers persists to facilitate early diagnosis, accurate prognosis, and treatment efficacy assessment.

Cuproptosis represents a distinct form of programmed cell death distinguished by the intracellular accumulation of copper, leading to subsequent disruption in mitochondrial lipid peroxidation and instability of Fe-S cluster proteins (10). The conversion of copper ions (Cu2+) to Cu by ferredoxin 1 (FDX1) facilitates the lipid acylation of mitochondrial proteins, thereby inducing the overproduction of crucial enzymes linked to the tricarboxylic acid (TCA) cycle. This mechanism plays a regulatory role in fundamental biological pathways, including the maintenance of redox balance, iron metabolism, oxidative phosphorylation, and the modulation of aberrant cell proliferation (11). Moreover, we observed that individuals diagnosed with active rheumatoid arthritis showed elevated levels of serum copper, exhibiting a negative correlation with hemoglobin levels, an ancillary disease marker, and a positive correlation with erythrocyte sedimentation rate and morning stiffness (12). Notably, a study proposed that serum copper levels could potentially serve as an indicator of erosive activity in RA (13). Numeric studies have proposed a multifaceted relationship between cuproptosis and RA (14–16). For example, Zhao et al. suggested that cuproptosis-related genes (PDHA1, PDHB, CDKN2A, and DLAT) were closely involved in the onset and development of RA (14). Jiang et al. (15) proposed a cuproptosis-related diagnostic model incorporating immune infiltration in RA. Hu et al. (16) developed a novel cuproptosis-related gene signature for RA. However, the current literature on the relationship between cuproptosis and RA remains relatively limited, highlighting the potential for cuproptosis to emerge as a new therapeutic target.

In this study, unveiling the ramifications of copper homeostasis imbalance on RA would significantly enhance our comprehension of its pathogenesis and facilitate the quest for efficacious agents. Cuproptosis-related genes are intricately associated with imbalance in copper homeostasis. To elucidate the repercussions of copper homeostasis imbalance on mitochondria in RA cells, we screened 10 DE-CRGs between RA samples and normal samples. Furthermore, to explore the impact of 10 DE-CRGs on RA, we examined the correlation between DE-CRGs and immune infiltration, immune factors, diagnostic efficacy, and predicted drugs. We assessed expression alterations in DE-CRGs across various RA cell clusters using single-cell RNA sequencing data. In summary, our findings provide valuable insights into characterizing copper homeostasis imbalance in RA and offer guidance for identifying potential therapeutic targets.

## Materials and methods

### Datasets

We collected the GSE93777 dataset (17) from the GEO database, which comprised 133 normal and 315 RA samples. Based on previous studies (18, 19), we selected 20 CRGs (FDX1, LIPT1, LIAS, DLD, PDHA1, PDHB, DLAT, SLC31A1, ATP7A, ATP7B, GLS, MTF1, CDKN2A, SLC25A3, GCSH, DBT, DLST, NLRP3, LIPT2, NFE2L2) for our analysis. Additionally, we obtained single-cell RNA sequencing data from serum transfer-induced inflammatory arthritis and RA tissue samples, as documented in the GSE129087 dataset (20).

### Differentially expressed cuproptosis-related genes

We applied log2 transformation to perform normalization, and utilized variance-stabilized counts to address the initial quality issues. To mitigate batch effects of samples, we employed the ‘limma’ package’s remove batch effect function (21). Initial steps include assessing the quality of the raw RNA-seq reads (e.g., using tools like FastQC) to ensure they are of high quality and suitable for downstream analysis. Limma is a popular tool that uses linear models and can incorporate batch effect correction by applying the duplicateCorrelation function, which models the correlation between technical replicates and corrects for batch effects. DE-CRGs between RA and normal groups were screen using the ‘limma’ package. The lmFit function in ‘limma’ package was employed to model the linear data, while the eBayes function estimated the model parameters. Thresholds for differentially expressed genes were set at adjusted *p*-value <0.01 and |logFC| > 0.5. The results were visualized through heatmaps and volcano plots using ‘pheatmap’ package and ‘ggplot2’ package. DE-CRGs were screened by the intersection between DEGs and CRGs. And DE-CRGs were depicted via a venn diagram generated using the ‘VennDiagram’ package.

### Functional analysis and PPI network analysis of DE-CRGs

To elucidate the potential biological functions of the DE-CRGs, we conducted GO and KEGG enrichment analyses. Utilizing the ‘clusterProfiler’ R package (22), we systematically annotated the DE-CRGs by employing the enrichGO and enrichKEGG functions for the enrichment analysis.

We utilized the STRING database^1^ to construct a PPI network of the DE-CRGs (23). The parameters of PPI network analysis: Organism: *Homo sapiens* (human). Interaction Score Threshold: A minimum interaction score of 0.4 (medium confidence) was used to filter potential protein interactions. Network type: We selected the “full network” option, which includes both experimentally determined interactions and predicted interactions. Edge length: We used the default edge length, which represents the strength of the interactions, with shorter edges indicating stronger associations. Additional Settings: We included both direct and indirect interactions to ensure a comprehensive understanding of the protein interaction network.

### Immune cell infiltration and immune correlation analysis of DE-CRGs

The CIBERSORT algorithm is used to ascertain the cellular composition of complex tissues based on their gene expression profiles (24). Thus, we applied the below algorithm to analyze the RNA-seq data obtained from both normal and RA tissues, facilitating the estimation of the relative proportions of immune-infiltrated cells. Subsequently, we utilized Pearson’s correlation analysis to evaluate the relationships between the DE-CRGs and immune cell, where statistical significance was set as *p*-value <0.05. We applied Bonferroni correction to account for multiple testing in the CIBERSORT results. Additionally, the correlation between DE-CRGs and various immune factors were retrieved from the tumor-immune system interaction database (TISIDB) (25). The parameters of the immune cell infiltration: Signature Matrix: We used the LM22 gene expression signature matrix, which is designed to deconvolve 22 human immune cell types, including T cells, B cells, monocytes, and neutrophils. Number of permutations: 1000 permutations were performed to estimate the significance of the results. *p*-value threshold: A p-value <0.05 was considered significant for the immune cell types showing differential infiltration. Absolute cell fractions: The analysis provides estimates of cell type proportions in the tissue, with a focus on assessing immune cell infiltration patterns.

### Clinical value of DE-CRGs in RA and therapeutic drug identification

To investigate the clinical relationships between DE-CRGs and RA, we used statistical analysis methods to compare between RA groups and normal groups. Receiver operating characteristic (ROC) curves were generated to assess diagnostic efficacy. ROC curve analysis was performed using the ‘pROC’ R package. Furthermore, we utilized Drug-Gene Interaction Databases (DGIdb and CMAP), which are online repositories detailing interactions between drugs and genes (26, 27), to predict potential therapeutic drugs associated with the DE-CRGs.

### scRNA sequencing data processing

The single-cell RNA sequencing dataset was derived from the serum transfer-induced inflammatory arthritis (STIA) model (GSE129087), which mimics synovial inflammation and joint pathology observed in human RA but does not fully recapitulate its autoimmune etiology (20). We used the ‘Seurat’ R package to perform data processing and cell grouping (28), and identified cell types by using the ‘SingleR’ package (29). Cell differentiation was analyzed using the ‘Monocle’ R package (30). We set the following thresholds: (i) Unique Molecular Identifier (UMI) Counts: Cells with UMI counts below 3 were excluded to eliminate low-quality cells. (ii) Gene Features: We filtered out cells expressing fewer than 200 and more than 4,000 genes to ensure sufficient gene coverage per cell. (iii) Mitochondrial Gene Expression: Cells exhibiting a mitochondrial gene expression ratio exceeding 5% were removed, as high mitochondrial content can indicate cell stress or apoptosis.

### Statistical analyses

The statistical analysis was performed utilizing R software (version 4.1.2). Group comparisons were executed using the Wilcoxon test. We used R packages: Seurat_4.3.0., SingleR_1.8.1, pROC_1.18.0, Monocle_2.34.0, CIBERSORT_0.1.0, limma_3.50.3, clusterProfiler_4.2.2, pheatmap_1.0.12, and ggplot2_3.4.2.

## Results

### Identification of DEGs and DE-CRGs

**Figure 1:**
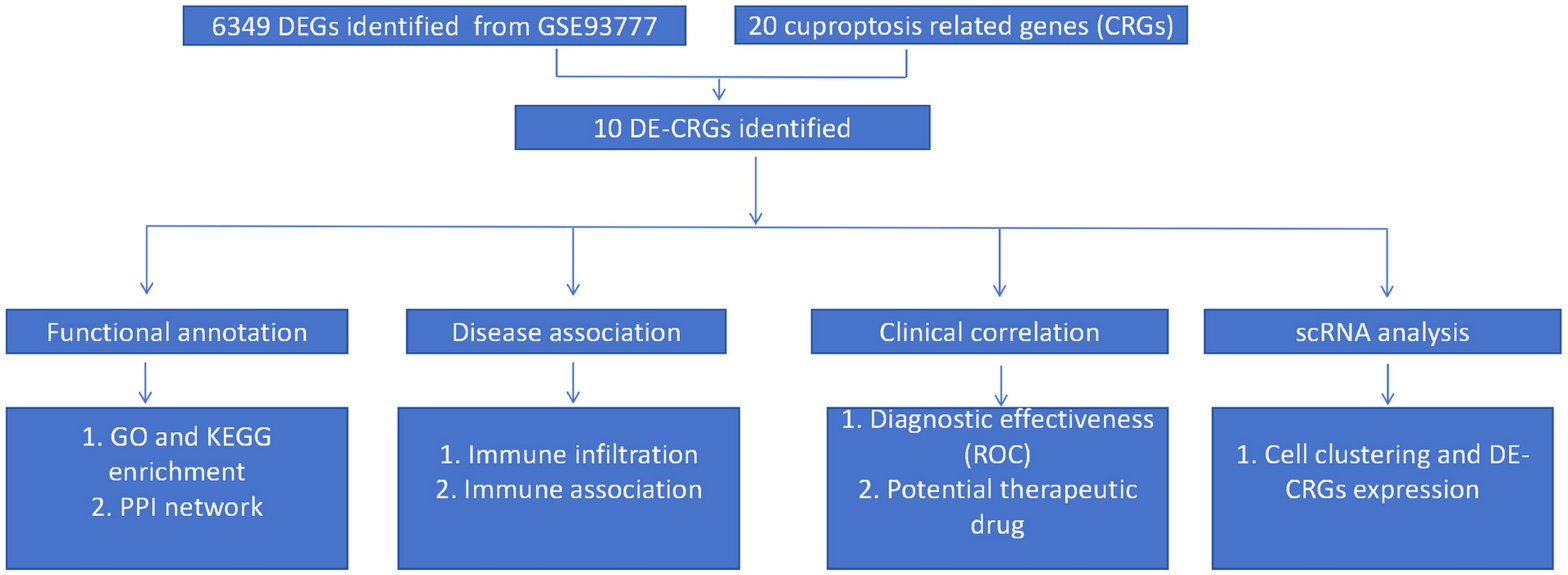
Flowchart of the study.

**Figure 2:**
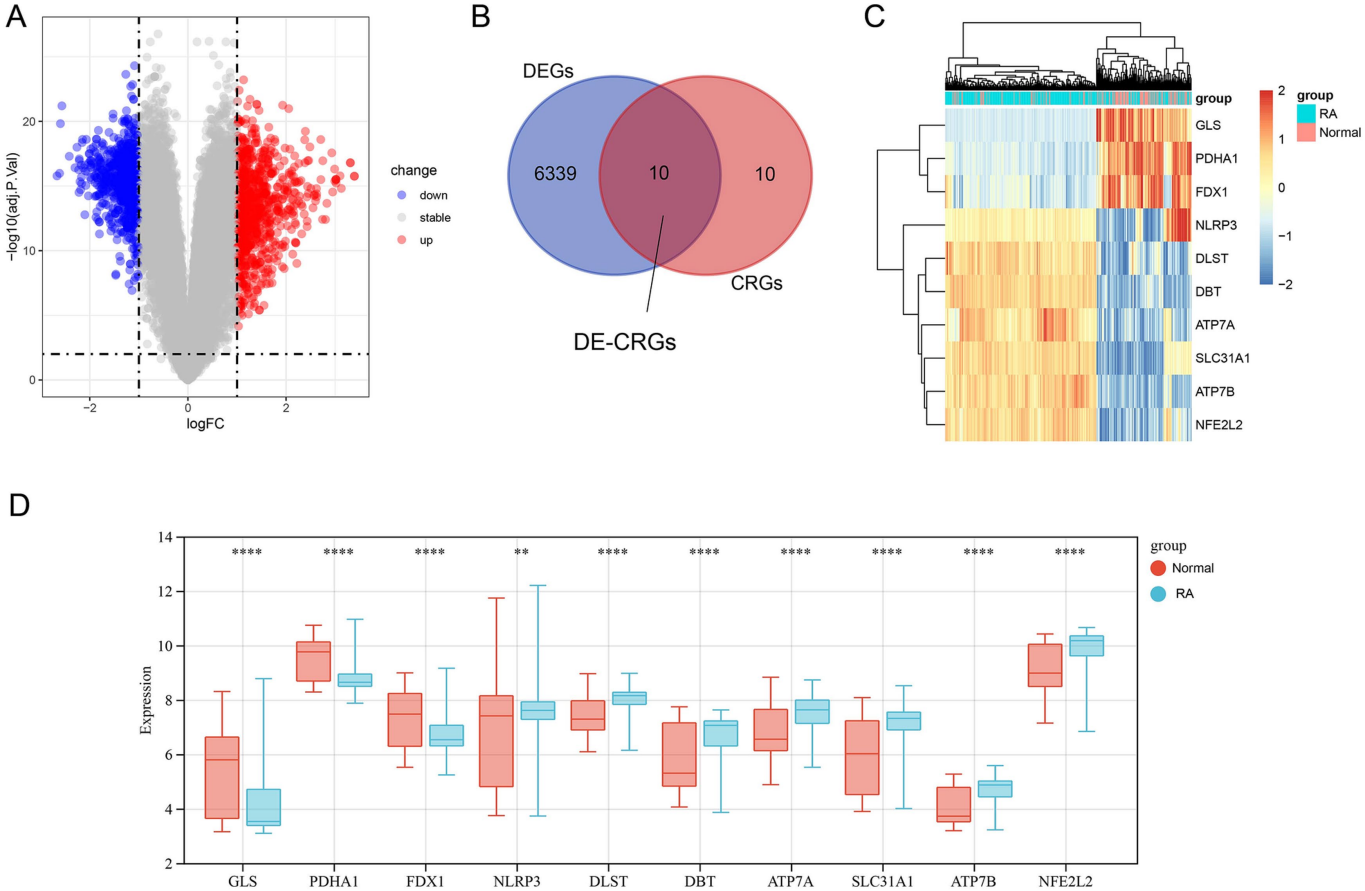
Screening of DEGs and DE-CRGs. **(A)** Volcano plot of DEGs. **(B)** Venn diagram representing the overlap between DEGs and CRGs. **(C)** Clustered heatmap of 10 DE-CRGs. **(D)** Differential expression analysis of 10 DE-CRGs between RA and normal tissues. ** *p* < 0.01, *** *p* < 0.001, **** *p* < 0.0001.

Figure 1 shows the overview of the study. We initially identified 6,349 differentially expressed genes (DEGs) using the ‘limma’ package (Figure 2A). Subsequently, 20 cuproptosis-related genes were matched with the DEGs, leading to the identification of 10 DE-CRGs relevant to RA tissue (Figure 2B). Among these, three DE-CRGs (GLS, PDHA1, and FDX1) exhibited downregulation, while seven DE-CRGs (NLRP3, DLST, ATP7B, ATP7A, NFE2L2, DBT, and SLC31A1) showed upregulation (Figures 2C,D).

### Function enrichment analysis of DE-CRGs

**Figure 3:**
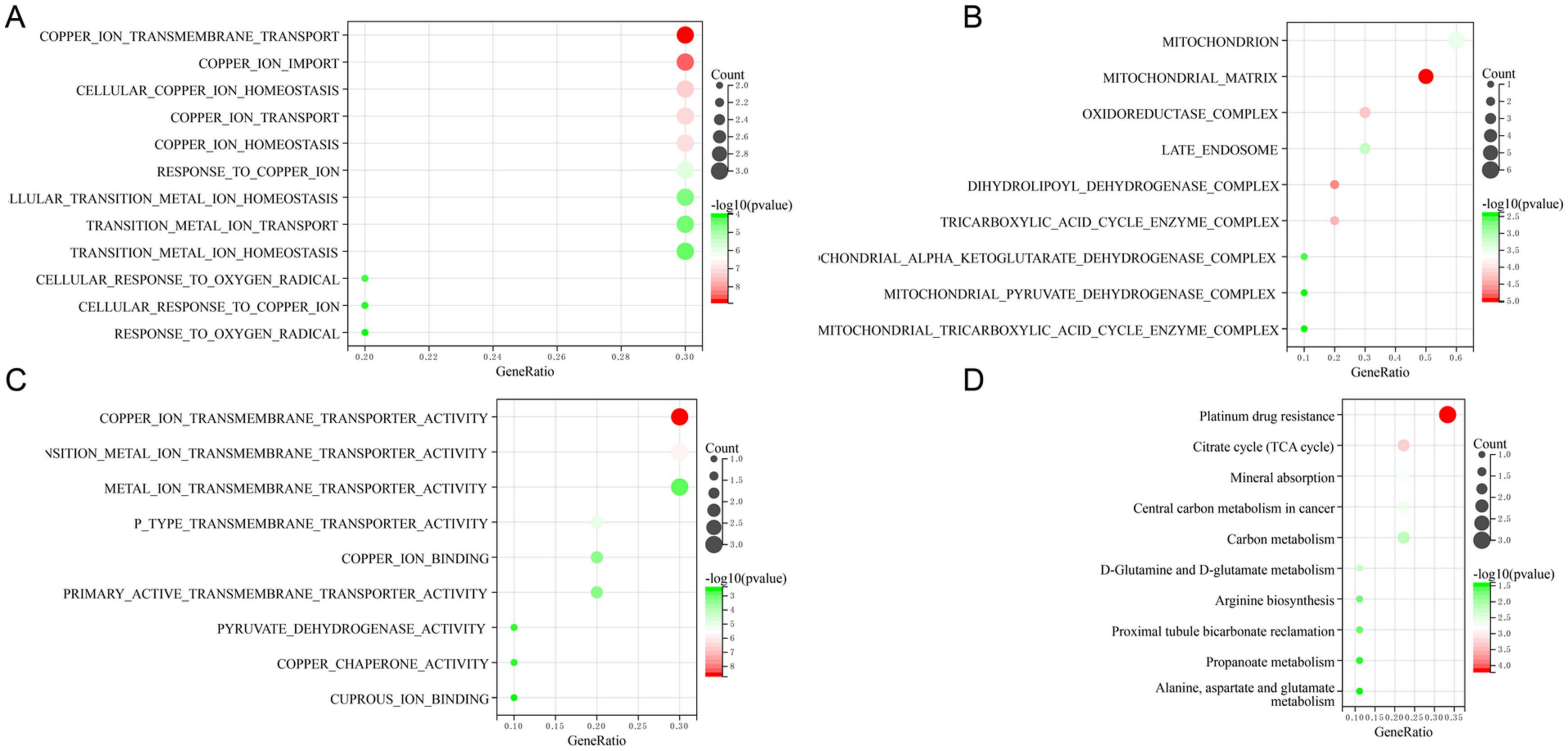
Function enrichment analysis of 10 DE-CRGs. **(A)** BP (biological process), **(B)** CC (cellular component), **(C)** MF (molecular function), **(D)** KEGG.

We utilized GO and KEGG enrichment analysis methods elucidate the potential biological functions associated with the 10 DE-CRGs. The GO analysis revealed that DE-CRGs were significantly enriched in several biological process (BP) terms, including copper ion transmembrane transport, copper ion import, cellular copper ion homeostasis, copper ion transport, and copper ion homeostasis (Figure 3A). Regarding cellular component (CC) terms, DE-CRGs were notably associated with mitochondrion, mitochondrial matrix, oxidoreductase complex, late endosome, and dihydrolipoyl dehydrogenase complex (Figure 3B). Furthermore, molecular function (MF) terms such as copper ion transmembrane transporter activity, transition metal ion transmembrane transporter activity, p type transmembrane transporter activity, and copper ion binding were significantly enriched among the DE-CRGs (Figure 3C).

The KEGG pathway analysis unveiled the involvement of DE-CRGs in a multitude of pathways, including platinum drug resistance, citrate cycle (TCA cycle), mineral absorption, central carbon metabolism in cancer, and carbon metabolism (Figure 3D). In summary, these findings revealed that DE-CRGs played crucial roles in a wide range of biological activities closely associated with mitochondrial function.

### PPI network analysis

**Figure 4:**
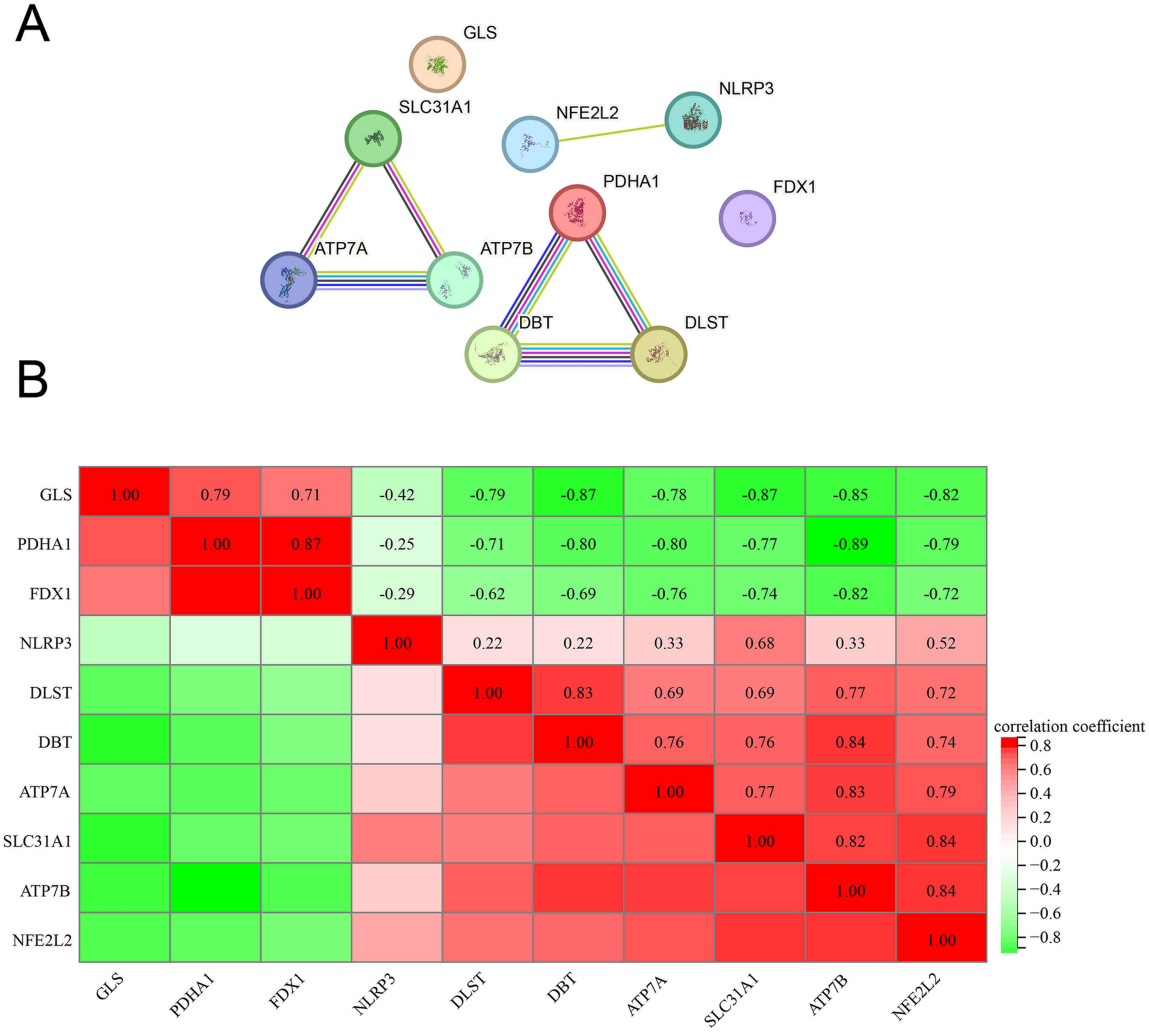
PPI analysis and Pearson correlation analysis. **(A)** PPI, **(B)** correlation analysis.

To delineate the interrelationships among the identified 10 DE-CRGs, we conducted PPI network analysis. Our findings revealed that ATP7A, ATP7B, and SLC31A1 exhibited close interconnectedness (Figure 4A), while PDHA1, DBT, and DLST also demonstrated significant interconnectivity. Moreover, NFE2L2 and NLRP3 were observed to interact within the network. Furthermore, we conducted Pearson correlation coefficient method to assess the correlation between the expression levels of 10 DEFGs. The results indicated predominantly positive correlations among most genes, with only a few displaying negative correlation relationships (Figure 4B).

### Immune cell infiltration and correlation analysis of DE-CRGs

**Figure 5:**
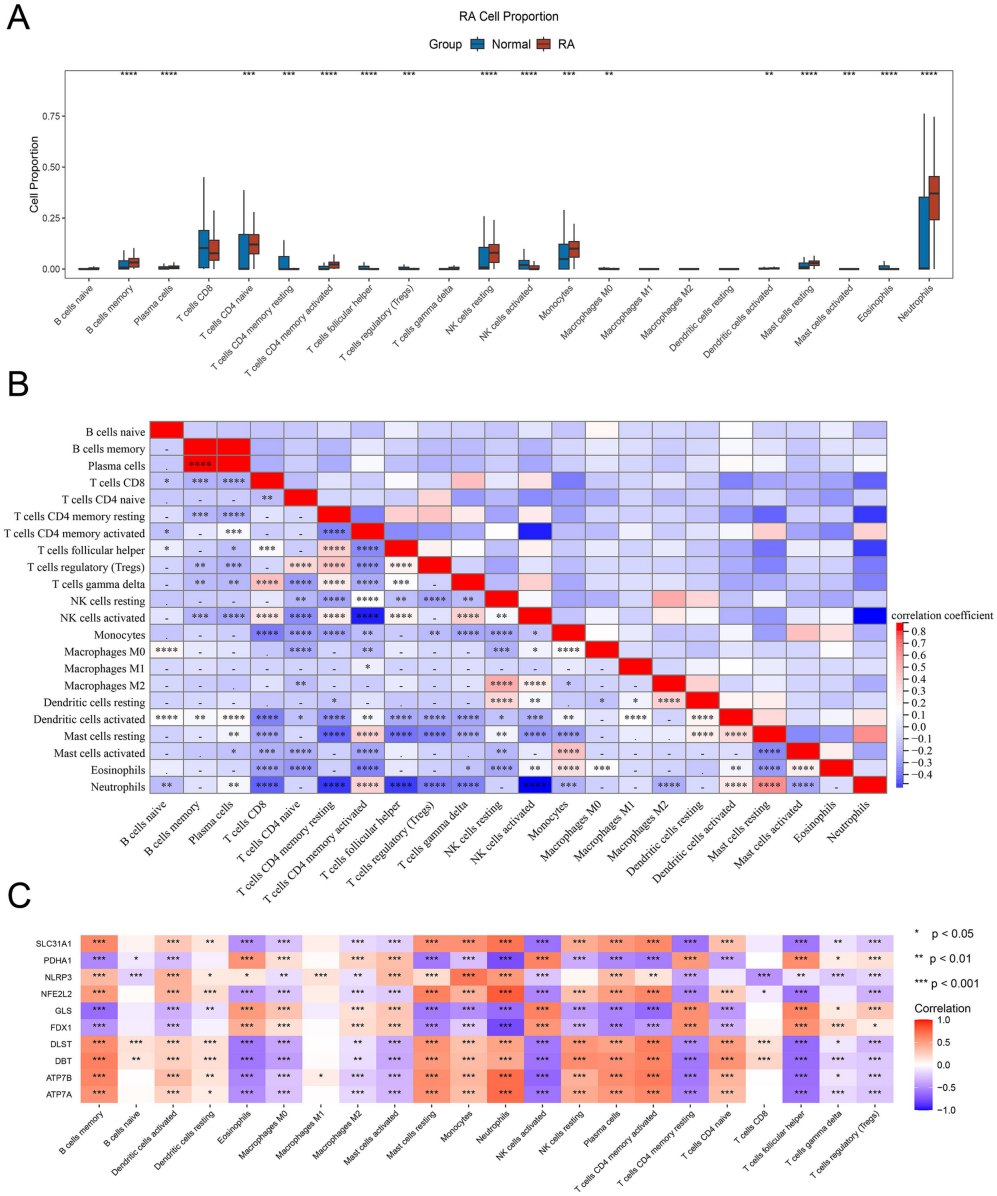
Immune infiltration of 10 DE-CRGs. **(A)** The fraction of immune cells comparison in RA and normal group. **(B)** Immune cells correlation map. **(C)** The correlation between DE-CRGs and immune cells. * *p* < 0.05, **p < 0.01, ***p < 0.001, ****p < 0.0001.

Given the common occurrence of immune system activation and response in RA, we employed the CIBERSORT algorithm to assess the proportion of 22 immune cell infiltrates within RA tissues. Our analysis unveiled notable alterations in immune cell composition between the RA and normal samples. Specifically, B cells memory, plasma cells, T cells CD4 naive, CD4+ T cells memory activated, NK cells resting, monocytes, mast cells resting, and neutrophils exhibited marked increases in the RA samples compared to the normal samples. Conversely, T cells CD4 memory resting, T cells follicular helper, Tregs, NK cells activated, and eosinophils were notably reduced in the RA samples (Figure 5A).

Numerous noteworthy correlations were discerned among the 22 immune cell types present in the RA tissue. For instance, a negative correlation was identified between neutrophils and T cells CD8, T cells CD4 memory activated, T cells follicular helper, Tregs, T cells gamma delta, and NK cells activated. Conversely, a positive correlation was observed between plasma cells and B cells memory, as well as between neutrophils and mast cells resting. Additionally, a positive correlation was detected between T cells gamma delta and T cells CD8, and between Macrophages M2 and NK cells resting (Figure 5B).

The identification of the association between DE-CRGs and immune cells aims to further elucidate the potential mechanism by which they impact the progression of RA. The results presented that some DE-CRGs were closely linked to immune cells. For example, SLC31A1, NLRP3, NFE2L2, DLST, DBT, ATP7B, and ATP7A showed a distinct positive correlation with B cells memory, mast cells resting, monocytes and neutrophils, but a negative correlation with macrophages M0, macrophages M2, NK cells resting, T cells CD4 memory resting, T cells follicular helper, T cells gamma delta and Tregs. PDHA1, GLS and FDX1 were positively correlated with eosinophils, NK cells resting, T cells CD4 memory resting, T cells follicular helper and negatively correlated with B cells memory, dendritic cells activated, mast cells resting, neutrophils, NK cells resting, plasma cells, T cells memory activated and T cells CD4 naive (Figure 5C).

**Figure 6:**
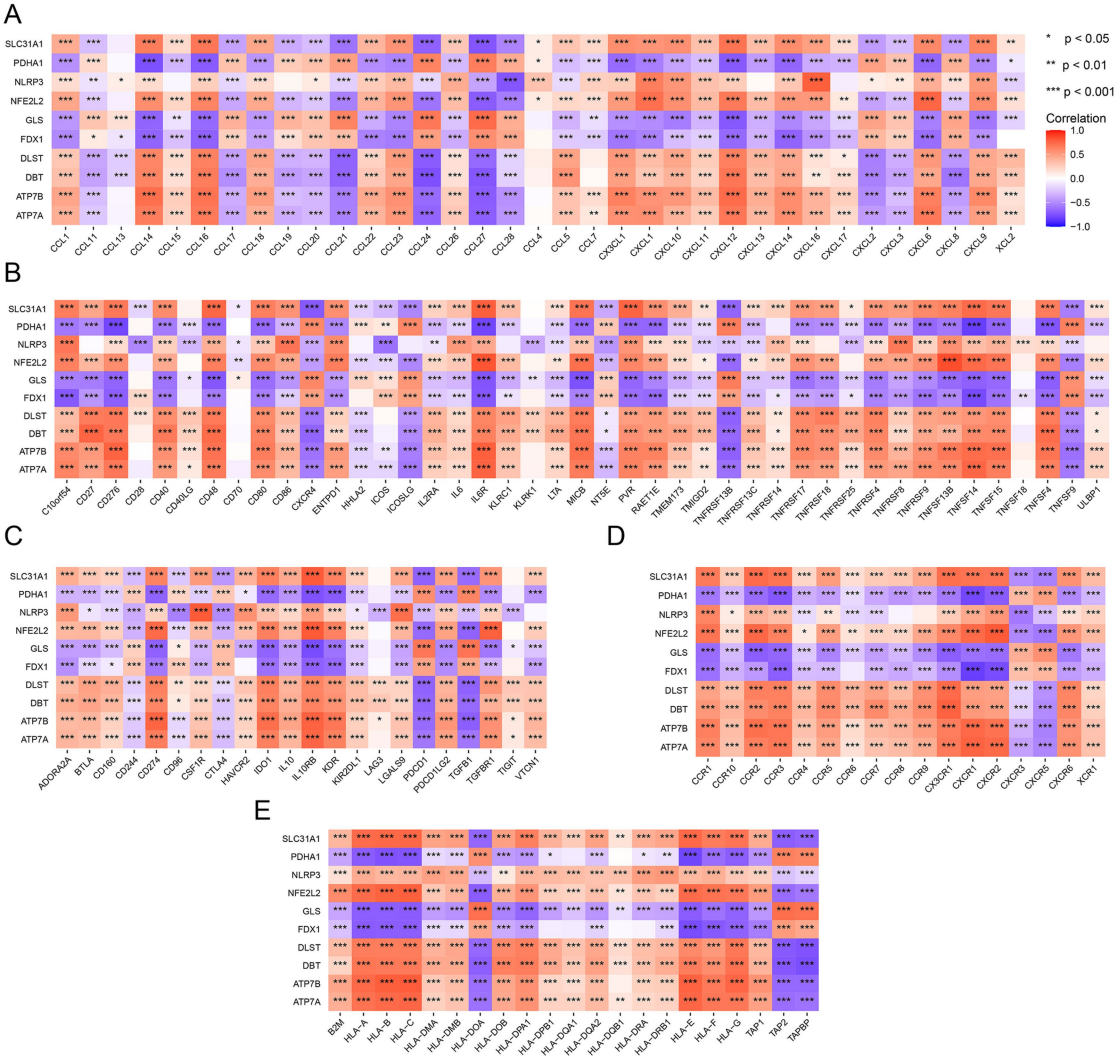
Immune-related analysis of DE-CRGs. **(A)** Correlation analysis between DE-CRGs and chemokine-related genes. **(B)** Correlation analysis between DE-CRGs and immunostimulator-related genes. **(C)** Correlation analysis between DE-CRGs and immunoinhibitor-related genes. **(D)** Correlation analysis between DE-CRGs and cell receptor-related genes. **(E)** Correlation analysis between DE-CRGs and MHC (major histocompatibility complex) related genes.

To further validate the relationship between the DE-CRGs and the immune system, we investigated their association with various immune factors, including chemokines, immunostimulators, immunoinhibitors, major histocompatibility complexes (MHCs), and cell receptors. Among the chemokine-related genes, SLC31A1 exhibited negative correlations with CCL21, CCL24, and CCL27, while showing positive correlations with CCL14, CCL16, and CXCL12 (Figure 6A). Regarding immunostimulatory-related genes, SLC31A1 demonstrated significant negative correlations with CXCR4, ICOSLG, TNFRSF13B, and TNFSF9, whereas it exhibited positive correlations with C10orf54, CD276, CD40, CD48, IL6R, MICB, PVR, TNFSF14, TNFSF15, and TNFSF4. PDHA1 was also notably negatively correlated with CD276, CD48, CD40, CD80, IL6R, MICB, PVR, RAET1E, TNFRSF17, TNFRSF18, TNFRSF4, TNFRSF8, TNFRSF9, TNFSF13B, TNFSF14, TNFSF15, and TNFSF4, while displaying positive correlations with CXCR4, ICOSLG, TNFRSF13B, and TNFSF9 (Figure 6B). Among the immunoinhibitory-related genes, NLRP3 exhibited significant negative correlations with CD96 and CTLA4, while GLS displayed significant positive correlations with PDCD1 and TGFB1. Similarly, FDX1 showed significant positive correlations with PDCD1 and TGFB1 (Figure 6C). In terms of cell receptor-related genes, DBT displayed marked negative correlations with CXCR3 and CXCR5, along with notable positive correlations with CCR1, CCR10, CCR2, CCR3, CCR5, CCR7, and CX3CR1 (Figure 6D). Furthermore, for MHC-related genes, ATP7A exhibited distinct negative correlations with HLA-DOA, TAP2, and TAPBP, while displaying distinct positive correlations with HLA-A, HLA-B, HLA-C, HLA-E, HLA-F, and HLA-G (Figure 6E). These analyses underscored the close relationship between the DE-CRGs and immune cell infiltration, underscoring their pivotal roles in modulating the immune response.

### Diagnostic effects of DE-CRGs

**Figure 7:**
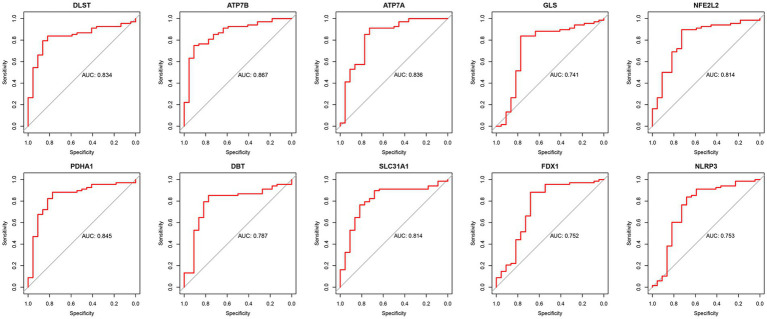
The ROC of ten DE-CRGs.

The diagnostic efficacy of the 10 DE-CRGs was assessed through ROC curve analysis, yielding the following AUC values: DLST: 0.834; ATP7B: 0.867; ATP7A: 0.836; GLS: 0.741; NFE2L2: 0.814; PDHA1: 0.845; DBT: 0.787; SLC31A1: 0.814; FDX1: 0.752; and NLRP3: 0.753 (Figure 7). These findings suggest that these 10 DE-CRGs hold significant promise as potential predictors of RA.

### Prediction of potential therapeutic drugs

To further elucidate the clinical relevance of the 10 DE-CRGs in RA, we conducted clinical drug predictive analysis using the DGIdb (26) and CMAP (27) databases. Our investigation revealed that most drugs targeting ATP7A, ATP7B, and SLC31A1 are anticancer agents, including thalidomide, progesterone, docetaxel, platinum-based drugs (oxaliplatin, carboplatin, cisplatin), daunorubicin, and cisplatin (Table 1). Another class of compounds, such as methotrexate, glutamine (Gln), and dexamethasone, was found to modulate GLS-mediated Gln catabolism by increasing substrate levels. Anakinra and MCC950 were identified as targeting NLRP3. Anakinra, a recombinant human interleukin-1 receptor antagonist, is used in RA (31). Guo et al. (32) highlighted the involvement of NLRP3 inflammasome in RA pathogenesis and suggested that targeting NLRP3 inflammasome with MCC950 could be a novel therapeutic strategy for RA. Additionally, drugs predicted to target the NFE2L2 gene, such as Lagascatriol, Andalusol, Irofulven, NK-252, RTA-408, and Sulforaphane, are commonly employed to alleviate RA inflammation in clinical practice. Moon et al. (33) demonstrated that sulforaphane treatment reduced arthritis severity and histologic inflammation in mice with collagen-induced arthritis (CIA). Choi et al. (34) found that sulforaphane inhibits synovial fibroblast proliferation, MMPs and COX-2 expression, and PGE2 production, suggesting its potential as a new therapeutic agent for RA. In summary, these results underscore the promise of developing novel drugs for RA treatment.

**Table 1:** Prediction of potential therapeutic drugs.

Drug	Gene	Source	PubChem ID	Ref
Thalidomide	ATP7A	DGIdb	92,142	(66)
Progesterone	ATP7A	DGIdb	5,994	(67)
Docetaxel	ATP7A	DGIdb	148,124	(68)
Platinum	ATP7B/ATP7A	DGIdb	23,939	(69)
Oxaliplatin	ATP7B/ATP7A	DGIdb	11,947,679	(69)
Carboplatin	ATP7B/ATP7A	DGIdb	73,554,252	(69, 70)
Daunorubicin	ATP7B	DGIdb	30,323	(71)
Cisplatin	ATP7B	DGIdb	86,820,626	(66)
Glutamine	GLS	DGIdb	6,992,086	(72–74)
Dexamethasone	GLS	DGIdb	5,743	(75)
Methotrexate	GLS	DGIdb	126,941	(76)
CB-839	GLS	CMAP	71,577,426	(77)
Carboplatin	SLC31A1	DGIdb	73,554,252	(78)
Cisplatin	SLC31A1	DGIdb	86,820,626	(79)
Anakinra	NLRP3	DGIdb	46,507,944	(80)
MCC950	NLRP3	CMAP	9,910,393	(81)
Lagascatriol	NFE2L2	DGIdb	10,448,831	(82)
Andalusol	NFE2L2	DGIdb	188,448	(82)
Irofulven	NFE2L2	DGIdb	148,189	(83)
NK-252	NFE2L2	CMAP	71,618,700	(84)
RTA-408	NFE2L2	CMAP	71,811,910	(85)
Sulforaphane	NFE2L2	CMAP	9,577,379	(86)
CPI-613	PDHA1	CMAP	70,881,528	(87)

### Single cell RNA sequencing data analysis of DE-CRGs

**Figure 8:**
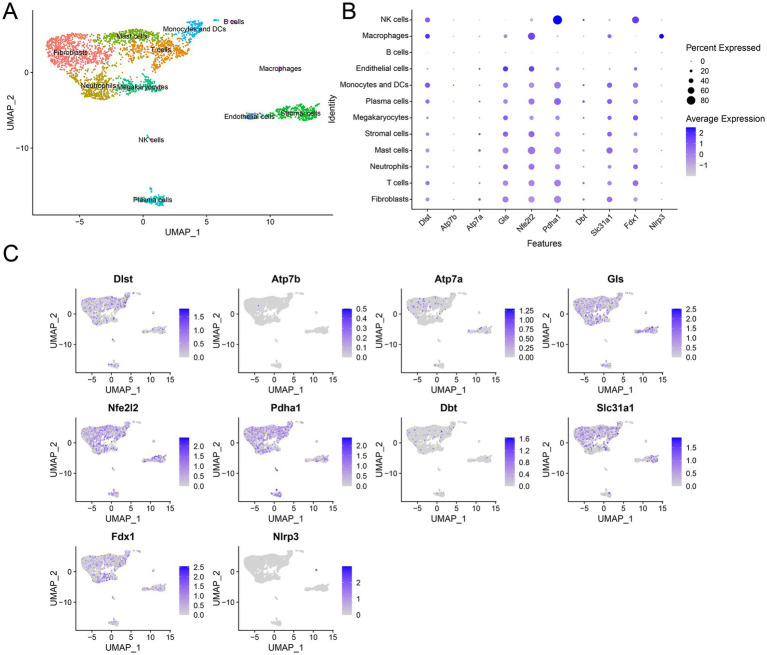
Single cell RNA analysis of DE-CRGs. **(A)** A UMAP pf 12 cell clusters. **(B)** Expression of DE-CRGs in different cell clusters. **(C)** Expression pattern of CRGs at the single-cell level in different cell clusters.

To unveil the transcriptome regulation within RA tissues at the single-cell level, we utilized single cell RNA sequencing data obtained from serum transfer-induced inflammatory arthritis to analyze expression of DE-CRGs in single cell. The RA cells exhibited considerable heterogeneity, and using the ‘singleR’ R package, we classified them into 12 clusters, including fibroblasts, T cells, neutrophils, mast cells, stromal cells, megakaryocytes, plasma cells, monocytes and DCs, endothelial cells, B cells, macrophages and NK cells, based on cell markers (Figure 8A) (35). Then, we investigated the expression profiles of CRGs across these cell clusters and observed that CRGs were prominently expressed in fibroblasts (Figure 8B). Overall, our findings suggest that DE-CRGs are generally upregulated across various cell clusters and display notable variations in fibroblasts (Figure 8C).

## Discussion

Cuproptosis has emerged as a novel mechanism of mitochondrial cell death triggered by disruptions in copper homeostasis. Increasing evidence from bioinformatics studies suggested that cuproptosis was involved in immune infiltration in numeric cancers, including clear cell renal cell carcinoma (36), head and neck squamous carcinomas (37), and lung adenocarcinoma (38). Cell death dysregulation plays a significant role in the initiation and development of RA, characterized by heightened chondrocyte apoptosis, diminished chondrocyte proliferation, and impaired extracellular matrix synthesis. However, the variation in copper content within RA tissue and specific RA cell clusters remains unclear. Given that cuproptosis stems from an imbalance in copper homeostasis driven by intracellular copper accumulation, we investigated this issue using nine DE-CRGs.

Our findings revealed a downregulation of GLS, PDHA1, and FDX1 expression, and an upregulation of NLRP3, DLST, ATP7B, ATP7A, NFE2L2, DBT, and SLC31A1 in whole RA tissues. SLC31A1 is involved in the transport of copper ions across cell membranes and plays a crucial role in maintaining cellular copper homeostasis. ATP7A is responsible for transporting copper ions across cell membranes and is involved in maintaining copper homeostasis within cells. Notably, both SLC31A1 and ATP7A are upregulated in response to elevated intracellular copper levels (39). DLST, an enzyme involved in the tricarboxylic acid (TCA) cycle, plays a crucial role in cellular metabolism by catalyzing the transfer of succinyl groups from succinyl-CoA to the enzyme lipoamide in the TCA cycle, facilitating the conversion of *α*-ketoglutarate to succinyl-CoA (40). However, the implications of these preliminary findings concerning copper reduction in whole RA tissue remained unclear. Therefore, we conducted further analysis on RA single-cell RNA sequencing (scRNA-seq) data to scrutinize the expression levels of the nine differentially expressed cuproptosis-related genes (DE-CRGs) across various cell clusters. Interestingly, we observed upregulated expression of all DE-CRGs, including DLST, SLC31A1, and GLS, in fibroblast and endothelial cell clusters. Conversely, minimal changes in the expression levels of the DE-CRGs were noted in other RA tissue cells, such as stem cells. GLS, an enzyme responsible for catalyzing the deamination of glutamine into glutamate, is a pivotal component of glutamine metabolism, serving as a fundamental energy source for proliferating cells (41, 42). Glutamine metabolism also plays a crucial role in various biological processes, including biosynthesis, antioxidant defense, and regulation of cell signaling (43). Moreover, glutamine induces a substantial carbon influx into the TCA cycle during cell proliferation. Therefore, the six identified differentially expressed cuproptosis-related genes (DE-CRGs) are intricately associated with protein lipoylation and energy metabolism processes within the mitochondria.

Subsequently, we scrutinized the biological functions, disease associations, and clinical correlations of the DE-CRGs, taking into account the tissue perspective. Our analyses, including GO and KEGG pathway analyses, unveiled the involvement of DE-CRGs in several critical pathways. Specifically, these pathways encompassed platinum drug resistance, the citrate cycle (TCA cycle), mineral absorption, central carbon metabolism in cancer, and carbon metabolism. The TCA cycle is crucial for cellular energy production and has been implicated in various inflammatory conditions. Alterations in mitochondrial function and energy metabolism are observed in RA, suggesting a potential link between TCA cycle dysregulation and RA pathogenesis (44). Mineral absorption, particularly of calcium and magnesium, plays a significant role in bone health. In RA patients, altered mineral metabolism can contribute to bone density loss and joint damage (45). Carbon metabolism, including the metabolism of glucose, fatty acids, and amino acids, is central to cellular function. In RA, dysregulated carbon metabolism contributes to inflammation and joint damage. Studies have identified metabolic changes in RA, such as increased glycolysis and altered fatty acid metabolism, which support the inflammatory processes characteristic of the disease (46, 47). Furthermore, our PPI analysis revealed that proteins sharing similar biological functions interacted closely with each other, potentially coordinating their roles in mediating essential cellular processes.

Our investigation into immune cell infiltration revealed significant alterations in various cell populations between the RA and normal groups. Specifically, B cells memory, plasma cells, T cells CD4 naive, CD4+ T cells memory activated, NK cells resting, monocytes, mast cells resting, and neutrophils exhibited elevated levels in the RA group compared to the normal group. Kishikawa et al. (48) demonstrated that the increased plasma nucleotide levels in RA patients. Lyu et al. (49) reported that CD305 reduction may mediate the excessive activation of memory CD4+ T cells and participate in the development of RA. Neutrophils play a crucial role in RA initiation and progression (50). After migrating into the articular cavity, they become activated, triggering inflammation (51). This leads to prolonged survival, excessive inflammatory activity, and increased oxidative stress, all of which contribute to RA pathogenesis (52). Conversely, T cells CD4 memory resting, T cells follicular helper, Tregs, NK cells activated, and eosinophils displayed decreased levels in the RA group. Sofi et al. (53) presented that eosinophilia occurred in several rheumatic diseases, including RA, and was associated with high disease activity and poor prognosis. Notably, we observed a negative correlation between activated and resting immune cells, while immune cells with similar functional states exhibited positive correlations. Plasma cells, arising from mature B lymphocytes, play a pivotal role in humoral immunity (54). Additionally, activated memory CD4+ T cells contribute to cellular immunity and collaborate with B cells (55). Neutrophils, as integral components of the innate immune system, act as frontline defenders and orchestrate subsequent adaptive immune responses (56). In summary, our findings suggest that RA triggers activation across humoral, cellular, and innate immune systems, which mutually reinforce each other. Furthermore, our analysis revealed a close association between differentially expressed cell regulatory genes (DE-CRGs) and resting immune cells, indicating their potential as novel targets for modulating excessive immune responses in RA.

We observed correlations between the 10 DE-CRGs and numeric immune factors. Particularly within the chemokine-related genes, our analysis revealed that SLC31A1 exhibited a negative correlation with CCL21, CCL24, and CCL27, while it displayed positive correlations with CCL14, CCL16, CXCL12, and CXCL14. Chemokines constitute a large protein family characterized by low molecular weight structures, and they play pivotal roles in regulating immune cell residence, migration, and inflammatory responses. The CC chemokine family primarily facilitates the recruitment of lymphocytes, whereas the CXC chemokine family is closely associated with neutrophils and monocytes (57). Additionally, CXCL12 and CXCL14 have been shown to augment macrophage function (58, 59). Therefore, our findings suggest that SLC31A1 is more inclined toward recruiting neutrophils and macrophages rather than lymphocytes.

Among the immunoinhibitory-related genes, we observed significant correlations. Specifically, SLC31A1 exhibited notably negative correlations with CXCR4, ICOSLG, TNFRSF13B, and TNFSF9, while it displayed positive correlations with C10orf54, CD276, CD40, CD48, IL6R, MICB, PVR, TNFSF14, TNFSF15, and TNFSF4. Similarly, PDHA1 demonstrated remarkable negative correlations with CD276, CD48, CD40, CD80, IL6R, MICB, PVR, RAET1E, TNFRSF17, TNFRSF18, TNFRSF4, TNFRSF8, TNFRSF9, TNFSF13B, TNFSF14, TNFSF15, and TNFSF4, while being positively correlated with CXCR4, ICOSLG, TNFRSF13B, and TNFSF9. The protein encoded by the CD276 gene belongs to the immunoglobulin superfamily and is believed to play a role in regulating T-cell-mediated immune responses (60). CD40, also known as tumor necrosis factor receptor superfamily member 5 (TNFRSF5), is a cell surface receptor protein found on antigen-presenting cells such as B cells, dendritic cells, and macrophages. It plays a crucial role in the immune system by providing co-stimulatory signals necessary for the activation of B cells and other immune cells (61). CD48 exhibits anti-inflammatory properties in *Staphylococcus aureus* Enterotoxin B-induced eosinophilic inflammation (62), while CXCR4 has been associated with the anti-inflammatory properties of mesenchymal stromal cells (63). Additionally, ICOS/ICOSL upregulation has been linked to inflammatory responses and endothelial dysfunction in type 2 diabetes mellitus (64). Our findings suggest that SLC31A1 and PDHA1 may be associated with anti-inflammatory effects based on these correlations.

In assessing the predictive potential of DE-CRGs in RA, our findings revealed ATP7B to exhibit the most robust diagnostic effect, followed by PDHA1 and DLST. The ATP7B gene encodes a protein called ATPase copper-transporting beta (ATP7B), which is primarily involved in transporting copper ions across cell membranes. This protein is crucial for maintaining copper homeostasis within the body by facilitating the incorporation of copper into ceruloplasmin and its subsequent secretion into the bloodstream (65). Despite its known role in copper transport, the precise impact of ATP7B on RA pathogenesis remains unclear. However, considering our results alongside those of previous investigations, PDHA1 and DLST emerge as candidates capable of disrupting intracellular copper homeostasis and mitochondrial function in the context of RA. Consequently, these findings may offer insights into novel etiological mechanisms and therapeutic targets for RA.

In the realm of predictive drugs for RA treatment, focusing on DE-CRGs, our analysis pinpointed several key targets, notably ATP7A, ATP7B, SLC31A1, GLS, and NFE2L2. The drugs identified for targeting ATP7A, ATP7B, and SLC31A1 primarily comprised anticancer agents, including thalidomide, progesterone, docetaxel, and various platinum-based chemotherapies such as oxaliplatin, carboplatin, daunorubicin, and cisplatin. Additionally, a subset of compounds, such as methotrexate, glutamine (Gln), and dexamethasone, were highlighted for their potential to mitigate the catabolism of Gln catalyzed by GLS by bolstering substrate levels. Of particular interest were drugs predicted to modulate the NFE2L2 gene, including Lagascatriol, Andalusol, Irofulven, NK-252, RTA-408, and Sulforaphane, which are commonly employed to alleviate inflammation associated with RA in clinical practice. These findings offer insights into novel candidates for systemic antimicrobial therapy. Furthermore, our analysis revealed upregulated expression of ATP7B and NFE2L2 in both RA tissues and cell clusters, suggesting that attenuating ATP7B decomposition may represent a promising avenue for future research into RA treatment.

This research has sparked numerous inquiries that warrant further exploration. A heightened emphasis on copper intake from daily essentials and diets could yield intriguing insights, enhancing our comprehension of the acquired induction and prevention of RA. Substantial efforts will be required to elucidate alterations in the transcription of DE-CRGs and subsequent post-translational modifications of coded proteins associated with mitochondrial dysfunction. Moreover, future studies could evaluate the potential therapeutic benefits of drugs targeting GLS, ATP7B, and ATP7A in the context of RA. Although the study has several limitations, particularly the lack of experimental validation, we plan to address these through follow-up research. This will include *in vitro* assays to explore the functional roles of ATP7A, ATP7B, and GLS in RA pathogenesis, as well as *in vivo* models to evaluate their therapeutic potential.

## Data Availability

Publicly available datasets were analyzed in this study. This data can be found at: GEO (https://www.ncbi.nlm.nih.gov/geo/), accession numbers GSE93777 and GSE129087.

## Footnotes

^1^ http://string-db.org/
