# Supplementary figures and images for: Identification of immune characteristic biomarkers and therapeutic targets in cuproptosis for rheumatoid arthritis by integrated bioinformatics analysis and single-cell RNA sequencing analysis
Source: Front Med (Lausanne). 2025 Mar 17;12:1520400. doi: 10.3389/fmed.2025.1520400 (PMC11955502; doi:10.3389/fmed.2025.1520400)

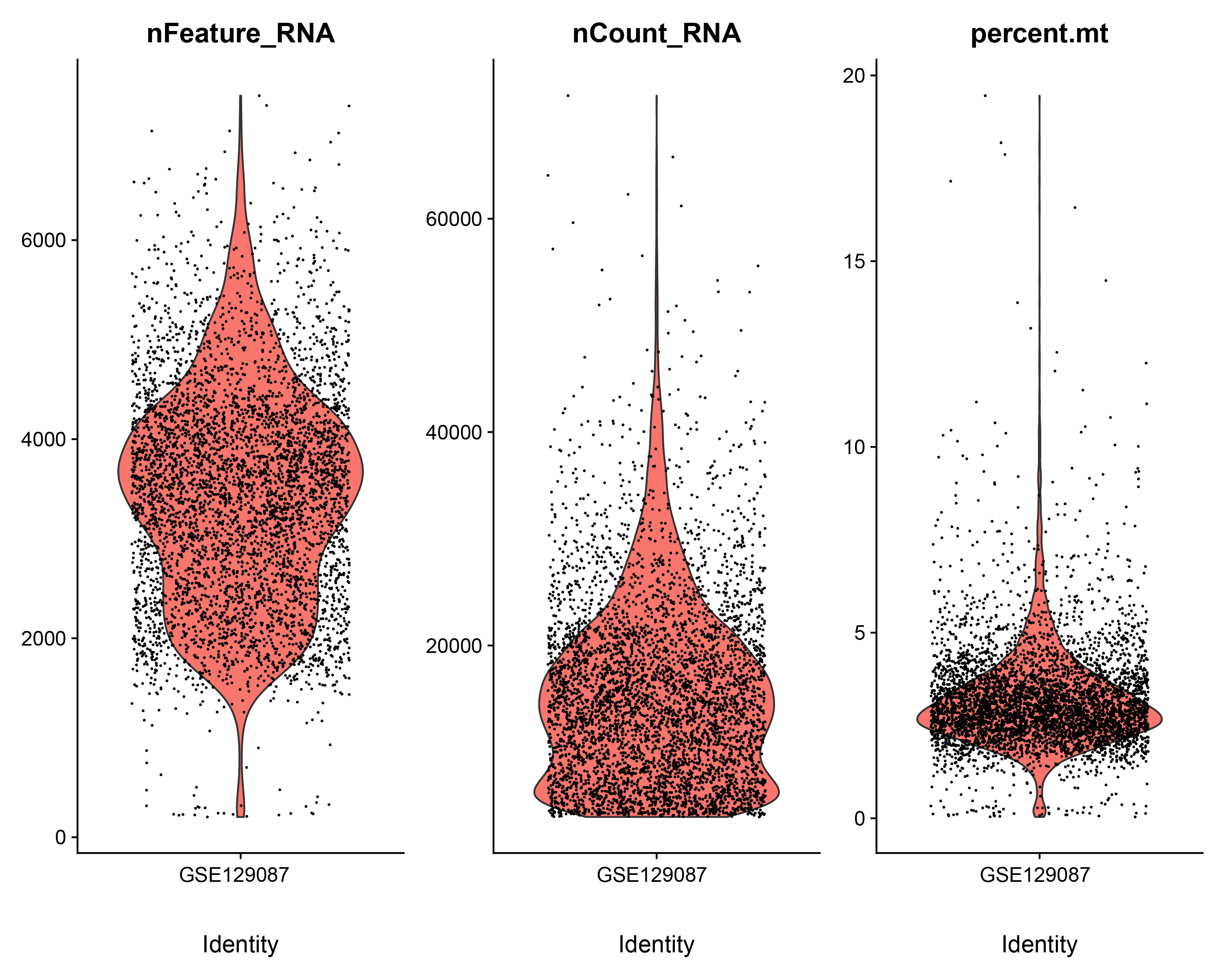

Supplement: Supplementary file 1 [file Image_1.JPEG]
